# Supplementary material for: Fault tolerance in distributed systems using deep learning approaches
Source: PLoS One. 2025 Jan 7;20(1):e0310657. doi: 10.1371/journal.pone.0310657 (PMC11706390; doi:10.1371/journal.pone.0310657)
Supplement: Attachment — Submitted filename: Response to Reviewers.pdf [file pone.0310657.s001.pdf]

PONE-D-24-09708

Fault Tolerance in Distributed Systems Using Deep Learning Approaches

PLOS ONE

Dear Dr. Abul Bashar

Thank you for your guiding comments which have been considered in the revised version of the manuscript.

1. Please ensure that your manuscript meets PLOS ONE's style requirements, including those for file naming. The PLOS ONE style templates can be found at..	Corrected as suggestion. Thanks for positive comments and feedback.
2. Please note that PLOS ONE has specific guidelines on code sharing for submissions in which author-generated code underpins the findings in the manuscript. In these cases, all author-generated code must be made available without restrictions upon publication of the work. Please review our guidelines at https://journals.plos.org/plosone/s/materials-and-software-sharing#loc-sharing-code and ensure that your code is shared in a way that follows best practice and facilitates reproducibility and reuse.	Corrected as suggestion. Three Python code files included with this file and are based upon requested. Thanks for positive comments and feedback.
3. Financial disclosure: Please state what role the funders took in the study.<br><br>Please include this amended Role of Funder statement in your cover letter; we will change the online submission form on your behalf.	Added to the financial disclosure statement. The funder covers the publication fees if it is published within specific time and in well-known ISI journal.
4. Please update your submission to use the PLOS LaTeX template.	Corrected as suggestion.
5. Data Availability Statement and ethical standard.	Corrected as suggestion.
6. Please amend your list of authors on the manuscript to ensure that each author is linked to an affiliation. Authors' affiliations should reflect the institution where the work was done.	Corrected as suggestion.
7. We note that Figure(s) 2 and 3 in your submission contain copyrighted images. All PLOS content is published under the Creative Commons Attribution License (CC BY 4.0), which means that the manuscript, images, and Supporting Information files will be freely	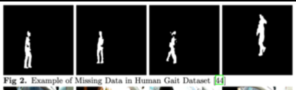<br>Fig 2. Example of Missing Data in Human Gait Dataset [44]<br>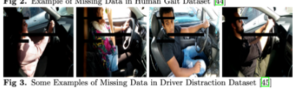<br>Fig 3. Some Examples of Missing Data in Driver Distraction Dataset [45]

available online, and any third party is permitted to access, download, copy, distribute, and use these materials in any way, even commercially, with proper attribution.	H. Eraqi, Y. Abouelnaga, M. Saad, M. Moustafa, "Driver Distraction Identification with an Ensemble of Convolutional Neural Networks", Journal of Advanced Transportation, Machine Learning in Transportation (MLT) Issue, 2019.<br><br>Y. Abouelnaga, H. Eraqi, and M. Moustafa. "Real-time Distracted Driver Posture Classification". Neural Information Processing Systems (NIPS 2018), Workshop on Machine Learning for Intelligent Transportation Systems, Dec. 2018. https://arxiv.org/abs/1706.09498<br><br>Wang, Liang, et al. "Silhouette analysis-based gait recognition for human identification." IEEE transactions on pattern analysis and machine intelligence 25.12 (2003): 1505-1518.<br><br>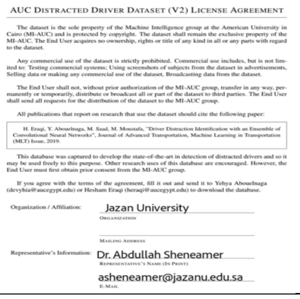<br>AUC DISTRACTED DRIVER DATASET (V2) LICENSE AGREEMENT<br><br>The dataset is the sole property of the Machine Intelligence group at the American University in Cairo (MI-AUC) and is protected by copyright. The dataset shall remain the exclusive property of the MI-AUC. The End User acquires no ownership, rights or title of any kind in all or any parts with regard to the dataset.<br><br>Any commercial use of the dataset is strictly prohibited. Commercial use includes, but is not limited to: Testing commercial systems; Using screenshots of subjects from the dataset in advertisements, Selling data or making any commercial use of the dataset, Broadcasting data from the dataset.<br><br>The End User shall not, without prior authorization of the MI-AUC group, transfer in any way, permanently or temporarily, distribute or broadcast all or part of the dataset to third parties. The End User shall send all requests for the distribution of the dataset to the MI-AUC group.<br><br>All publications that report on research that use the dataset should cite the following paper:<br><br>H. Eraqi, Y. Abouelnaga, M. Saad, M. Moustafa, "Driver Distraction Identification with an Ensemble of Convolutional Neural Networks", Journal of Advanced Transportation, Machine Learning in Transportation (MLT) Issue, 2019.<br><br>This database was captured to develop the state-of-the-art in detection of distracted drivers and so it may be used freely to this purpose. Other research uses of this database are encouraged. However, the End User must first obtain prior consent from the MI-AUC group.<br><br>If you agree with the terms of the agreement, fill it out and send it to Yehya Abouelnaga (devyhia@aucegypt.edu) or Hesham Eraqi (heraqi@aucegypt.edu) to download the database.<br><br>Organization / Affiliation: Jazan University<br>ORGANIZATION<br>MAILING ADDRESS<br>Representative's Information: Dr. Abdullah Sheneamer<br>REPRESENTATIVE'S NAME (IN PRINT)<br>asheneamer@jazanu.edu.sa<br>E-MAIL

PONE-D-24-09708

Fault Tolerance in Distributed Systems Using Deep Learning Approaches

PLOS ONE

Dear Dr. Sheneamer,

Thank you for submitting your manuscript to PLOS ONE. After careful consideration, we feel that it has merit but does not fully meet PLOS ONE's publication criteria as it currently stands. Therefore, we invite you to submit a revised version of the manuscript that addresses the points raised during the review process.

Please submit your revised manuscript by May 10 2024 11:59PM. If you will need more time than this to complete your revisions, please reply to this message or contact the journal office at plosone@plos.org. When you're ready to submit your revision, log on to https://www.editorialmanager.com/pone/ and select the 'Submissions Needing Revision' folder to locate your manuscript file.

Please include the following items when submitting your revised manuscript:

- A rebuttal letter that responds to each point raised by the academic editor and reviewer(s). You should upload this letter as a separate file labeled 'Response to Reviewers'.
- A marked-up copy of your manuscript that highlights changes made to the original version. You should upload this as a separate file labeled 'Revised Manuscript with Track Changes'.
- An unmarked version of your revised paper without tracked changes. You should upload this as a separate file labeled 'Manuscript'.

If you would like to make changes to your financial disclosure, please include your updated statement in your cover letter. Guidelines for resubmitting your figure files are available below the reviewer comments at the end of this letter.

If applicable, we recommend that you deposit your laboratory protocols in protocols.io to enhance the reproducibility of your results. Protocols.io assigns your protocol its own identifier (DOI) so that it can be cited independently in the future. For instructions see: https://journals.plos.org/plosone/s/submission-guidelines#loc-laboratory-protocols. Additionally, PLOS ONE offers an option for publishing peer-reviewed Lab Protocol articles, which describe protocols hosted on protocols.io. Read more information on sharing protocols at https://plos.org/protocols?utm_medium=editorial-email&utm_source=authorletters&utm_campaign=protocols.

We look forward to receiving your revised manuscript.

Kind regards,

Abul Bashar
Academic Editor
PLOS ONE

Journal requirements:

When submitting your revision, we need you to address these additional requirements.

1. Please ensure that your manuscript meets PLOS ONE's style requirements, including those for file naming. The PLOS ONE style templates can be found at

https://journals.plos.org/plosone/s/file?id=wjVg/PLOSOne_formatting_sample_main_body.pdf and

https://journals.plos.org/plosone/s/file?id=ba62/PLOSOne_formatting_sample_title_authors_affiliations.pdf

2. Please note that PLOS ONE has specific guidelines on code sharing for submissions in which author-generated code underpins the findings in the manuscript. In these cases, all author-generated code must be made available without restrictions upon publication of the work. Please review our guidelines at https://journals.plos.org/plosone/s/materials-and-software-sharing#loc-sharing-code and ensure that your code is shared in a way that follows best practice and facilitates reproducibility and reuse.

3. Thank you for stating the following financial disclosure:

[The authors extend the appreciation to the Deputyship for Research Innovation, Ministry of Education in Saudi Arabia for funding this research work through project number:ISP-2024].

Please state what role the funders took in the study. If the funders had no role, please state: "The funders had no role in study design, data collection and analysis, decision to publish, or preparation of the manuscript."

If this statement is not correct you must amend it as needed.

Please include this amended Role of Funder statement in your cover letter; we will change the online submission form on your behalf.

4. Please update your submission to use the PLOS LaTeX template. The template and more information on our requirements for LaTeX submissions can be found at http://journals.plos.org/plosone/s/latex.

5. We note that your Data Availability Statement is currently as follows: [We use some standardized datasets and they are cited]

Please confirm at this time whether or not your submission contains all raw data required to replicate the results of your study. Authors must share the “minimal data set” for their submission. PLOS defines the minimal data set to consist of the data required to replicate all study findings reported in the article, as well as related metadata and methods (https://journals.plos.org/plosone/s/data-availability#loc-minimal-data-set-definition).

For example, authors should submit the following data:

- The values behind the means, standard deviations and other measures reported;

- The values used to build graphs;

- The points extracted from images for analysis.

Authors do not need to submit their entire data set if only a portion of the data was used in the reported study.

If your submission does not contain these data, please either upload them as Supporting Information files or deposit them to a stable, public repository and provide us with the relevant URLs, DOIs, or accession numbers. For a list of recommended repositories, please see https://journals.plos.org/plosone/s/recommended-repositories.

If there are ethical or legal restrictions on sharing a de-identified data set, please explain them in detail (e.g., data contain potentially sensitive information, data are owned by a third-party organization, etc.) and who has imposed them (e.g., an ethics committee). Please also provide contact information for a data access committee, ethics committee, or other institutional body to which data requests may be sent. If data are owned by a third party, please indicate how others may request data access.

6. Please amend your list of authors on the manuscript to ensure that each author is linked to an affiliation. Authors’ affiliations should reflect the institution where the work was done (if authors moved subsequently, you can also list the new affiliation stating “current affiliation:….” as necessary).

7. We note that Figure(s) 2 and 3 in your submission contain copyrighted images. All PLOS content is published under the Creative Commons Attribution License (CC BY 4.0), which means that the manuscript, images, and Supporting Information files will be freely available online, and any third party is permitted to access, download, copy, distribute, and use these materials in any way, even commercially, with proper attribution. For more information, see our copyright guidelines: http://journals.plos.org/plosone/s/licenses-and-copyright.

We require you to either (1) present written permission from the copyright holder to publish these figures specifically under the CC BY 4.0 license, or (2) remove the figures from your submission:

A. You may seek permission from the original copyright holder of Figure(s) 2 and 3 to publish the content specifically under the CC BY 4.0 license.

We recommend that you contact the original copyright holder with the Content Permission Form (http://journals.plos.org/plosone/s/file?id=7c09/content-permission-form.pdf) and the following text:

“I request permission for the open-access journal PLOS ONE to publish XXX under the Creative Commons Attribution License (CCAL) CC BY 4.0 (http://creativecommons.org/licenses/by/4.0/). Please be aware that this license allows unrestricted use and distribution, even commercially, by third parties. Please reply and provide explicit written permission to publish XXX under a CC BY license and complete the attached form.”

Please upload the completed Content Permission Form or other proof of granted permissions as an "Other" file with your submission.

In the figure caption of the copyrighted figure, please include the following text: “Reprinted from [ref] under a CC BY license, with permission from [name of publisher], original copyright [original copyright year].”

B. If you are unable to obtain permission from the original copyright holder to publish these figures under the CC BY 4.0 license or if the copyright holder’s requirements are incompatible with the CC BY 4.0 license, please either i) remove the figure or ii) supply a replacement figure that complies with the CC BY 4.0 license. Please check copyright information on all replacement figures and update the figure caption with source information. If applicable, please specify in the figure caption text when a figure is similar but not identical to the original image and is therefore for illustrative purposes only

Additional Editor Comments

Please submit the paper in the required PLOS ONE format.

[Note: HTML markup is below. Please do not edit.]
:

[NOTE: If reviewer comments were submitted as an attachment file, they will be attached to this email and accessible via the submission site. Please log into your account, locate the manuscript record, and check for the action link "View Attachments". If this link does not appear, there are no attachment files.]

While revising your submission, please upload your figure files to the Preflight Analysis and Conversion Engine (PACE) digital diagnostic tool, https://pacev2.apexcovantage.com/. PACE helps ensure that figures meet PLOS requirements. To use PACE, you must first register as a user. Registration is free. Then, login and navigate to the UPLOAD tab, where you will find detailed instructions on how to use the tool. If you encounter any issues or have any questions when using PACE, please email PLOS at figures@plos.org. Please note that Supporting Information files do not need this step.
