# Supplementary material for: Fault tolerance in distributed systems using deep learning approaches
Source: PLoS One. 2025 Jan 7;20(1):e0310657. doi: 10.1371/journal.pone.0310657 (PMC11706390; doi:10.1371/journal.pone.0310657)
Supplement: Attachment — Submitted filename: Review_Responses_R2.pdf [file pone.0310657.s002.pdf]

Responses to reviewer 1

First of all, we would like to thank you very much for your valuable and fruitful comments and suggestions, that are taken as a guide to improve this work.

Comment	Response
The abstract is to be quantified. The highlights are not properly noted.	Updated, the main highlights are noted, including the use of deep learning for fault tolerance and recovery in three major scenarios.<br>The abstract now is quantified. The accuracies of the models are measured and the abstract shows the accuracy results.<br>Thanks for your valuable comments.
The limitations of previous works are to be included.	Updated, in the related work we first highlight previous works of the distributed system works. Then, the fault tolerance concept. After that, the use of machine learning in fault tolerance and finally the use of deep learning in fault tolerance.<br>At the end of first and second paragraph, in paragraph four as well, we show the limitations of the previous works and how we handle such limitation. For example, the traditional distributed system and fault tolerance do not use intelligent tools such as deep learning. In addition, they do not use deep learning for recovery, and to handle missing, corrupted and unrelated input.<br>Thanks for your valuable comments.
Section 2 and 3 needs improvement.	Section 2 and 3 have been improved. The updates are highlighted.<br>Thanks for your valuable comments.
Discussion part is poorly presented.	Updated, see sections 3, 4 and 6. The discussion has been improved.<br>Actually, to avoid the overlength of the paper, there is no specific section of

	discussion. However, the discussion of the main idea is included in Section 3, while the evaluation of our model and experiment is discussed within Section 4, and the limitations and threat of validity is presented in Section 6.<br><br>Thanks for your valuable comments.
Conclusion may be modified.	The conclusion had been modified.<br><br>Thanks for your valuable comments.
The limitations and time loss analysis may be included.	Updated, now it is included in the section of Limitation and Threat of Validity<br><br>Fault Tolerance Scope: Employing intelligent techniques such as deep learning techniques in the distributed system and fault recovery is an innovative task, without deep history or approved benchmark. Actually, deep learning models are effective in managing certain types of faults within distributed systems. However, their approach may not cover all potential fault scenarios, especially those involving complex, interconnected faults that can propagate across various system components.<br><br>Dataset Dependence: Find an existing suitable dataset to test the proposed idea is another challenge. In fact, the models' performance is heavily reliant on the quality and nature of the datasets they are trained on. For example, models trained on specific structured or unstructured data types might not perform optimally when applied to different data types. Therefore, train our models on both structured and unstructured datasets.<br><br>Fault Types: Examine different kinds of faults is another issue, where we investigate different kinds of faults, using three scenarios.<br><br>Fault Ratio: Faults ratios is another critical issue to investigate. Our work extends the experiment to test different faults ratios.

	Moreover, in cases involving larger faults, the recovery time could be considerable.<br><br>Computational Overhead: Implementing deep learning models for real-time fault detection and correction introduces computational overhead, which could be problematic for systems with stringent latency requirements. For example, the time taken for a model to make predictions during the fault recovery process can also be a bottleneck, especially in real-time systems where rapid decision-making is essential.<br><br>Training Time: Training deep learning models, particularly with large datasets or complex architectures like VGG16, VGG19, or ResNet34, is time-intensive. This significant time investment must be taken into account when deploying these models in practical applications.<br><br>Additionally, finding proper deep learning techniques and to generalize our findings are vital points to consider.
Bound condition for all equations are to be included.	Updated, see section 4, all equation results are between 0 and 1, as all variables are positive and the numerator is smaller than the denominator
1. If the authors have adequately addressed your comments raised in a previous round of review and you feel that this manuscript is now acceptable for publication, you may indicate that here to bypass the "Comments to the Author" section, enter your conflict of interest statement in the "Confidential to Editor" section, and submit your "Accept" recommendation.<br><br>Reviewer #1: All comments have been addressed	Thanks for your valuable comments
2. Is the manuscript technically sound, and do the data support the conclusions?	The technical part has been improved in all parts of the paper. Thanks for your valuable comments

The manuscript must describe a technically sound piece of scientific research with data that supports the conclusions. Experiments must have been conducted rigorously, with appropriate controls, replication, and sample sizes. The conclusions must be drawn appropriately based on the data presented.<br>Reviewer #1: Partly	
3. Has the statistical analysis been performed appropriately and rigorously?<br><br>Reviewer #1: N/A	-
4. Have the authors made all data underlying the findings in their manuscript fully available?<br><br>The PLOS Data policy requires authors to make all data underlying the findings described in their manuscript fully available without restriction, with rare exception (please refer to the Data Availability Statement in the manuscript PDF file). The data should be provided as part of the manuscript or its supporting information, or deposited to a public repository. For example, in addition to summary statistics, the data points behind means, medians and variance measures should be available. If there are restrictions on publicly sharing data—e.g. participant privacy or use of data from a third party—those must be specified.<br>Reviewer #1: Yes	Thanks for your valuable comments
5. Is the manuscript presented in an intelligible fashion and written in standard English?<br><br>PLOS ONE does not copyedit accepted manuscripts, so the language in submitted articles must be clear, correct, and unambiguous. Any typographical or grammatical errors should be corrected at revision, so please note any specific errors here.<br>Reviewer #1: Yes	Thanks for your valuable comments
